# A Rapid Method for the Simultaneous Analysis of Tocopherols and Tocopherol Quinones in Edible Oils Using Normal-Phase High-Performance Liquid Chromatography

**DOI:** 10.3390/foods15101767

**Published:** 2026-05-17

**Authors:** Hongyan Guo, Zengde Zhang, Sameh A. Korma, Taha Mehany, Tao Zhang, Liyou Zheng

**Affiliations:** 1 College of Biology and Food Engineering, Anhui Polytechnic University, Wuhu 241000, China; 2106032@mail.ahpu.edu.cn (H.G.); zhangzengde@outlook.com (Z.Z.); 2 Department of Food Science, Faculty of Agriculture, Zagazig University, Zagazig 44519, Egypt; sameh9251@scut.edu.cn; 3 School of Food Science and Engineering, South China University of Technology, Guangzhou 510641, China; 4 Food Technology Department, Arid Lands Cultivation Research Institute, City of Scientific Research and Technological Applications, Alexandria 21934, Egypt; tmehany@srtacity.sci.eg; 5 College of Food Science and Technology, Huazhong Agricultural University, Wuhan 430070, China

**Keywords:** tocopherol, tocopherol quinones, HPLC, simultaneous analysis, oil samples, method validation

## Abstract

A normal-phase high-performance liquid chromatography (HPLC) method with UV spectrophotometric detector (SPD) detection was established for the simultaneous analysis of four tocopherol (TP) homologues and their corresponding tocopherol quinones (TQs). Separation was performed on a Sepax Technologies silica column (4.6 mm × 250 mm, 5 μm) using a mobile phase consisting of *n*-hexane, isopropanol, and tetrahydrofuran, allowing the eight target compounds to be effectively separated within 15 min. Method validation was conducted using standard solutions prepared in *n*-hexane, providing a direct evaluation of the chromatographic performance under a relatively clean solvent system. The method showed satisfactory linearity, with R^2^ values of 0.9850–0.9996. The limit of detection (LOD) and limit of quantification (LOQ) ranges were 0.140–0.371 and 0.467–1.235 μg/mL for TP homologues and 0.0564–0.0856 and 0.1708–0.2595 μg/mL for TQ homologues, respectively. Precision was acceptable, with intra-day and inter-day relative standard deviations (RSD) below 2.69% and 3.78%, respectively. To further evaluate its applicability in oil matrices, recovery experiments were performed in peanut oil and camellia oil, yielding recoveries of 91.7–105.8% and 90.9–97.7% for TP homologues and 82.8–98.7% and 79.5–101.8% for TQ homologues, respectively. The method was further applied to edible oil samples. The results showed that the major TP homologues and detectable TQ homologues were determined, while some homologues were below the detection limits. Overall, the proposed method offers a simple and practical approach for the simultaneous analysis of TP and TQ homologues, thereby supporting further investigation of tocopherol oxidation in edible oils.

## 1. Introduction

Lipid oxidation remains a major challenge for the food industry due to its complexity and its substantial impact on oil quality, nutritional value, and shelf life [1]. The oxidative deterioration of edible oils is influenced by multiple factors, such as temperature, light exposure, oxygen concentration, and fatty acid composition [2]. During oxidation, various volatile secondary products, such as aldehydes and ketones, are formed, leading to undesirable flavors and reduced nutritional quality. Moreover, some lipid oxidation products may disturb the redox balance in vivo, posing potential health risks [1,3]. Food processing, like oilseed heat pretreatment, oil refining processes and home cooking, can further accelerate oxidative deterioration by depleting endogenous antioxidants naturally present in oils. Consequently, lipid oxidation not only compromises product quality but also causes considerable economic losses to the food industry [4]. Although synthetic antioxidants such as tert-butylhydroquinone and butylated hydroxytoluene are widely applied to retard oxidation, increasing consumer concern regarding the safety and acceptability of synthetic additives has driven growing interest in natural antioxidants and their behavior in edible oils [4].

Tocopherols (TPs), collectively referred to as vitamin E, are naturally occurring lipid- soluble antioxidants widely distributed in vegetable oils [5]. Structurally, TPs consist a chromanol ring and a phytyl side chain. They are classified into four homologues, namely α-, β-, γ-, and δ-TP, mainly based on the number and position of methyl groups on the chromanol ring [6]. TPs play a critical role in protecting lipids, particularly polyunsaturated fatty acids, from oxidative degradation [7]. Their antioxidant activity primarily arises from scavenging reactive oxygen species and donating hydrogen from the phenolic hydroxyl group to lipid radicals, thereby inhibiting the propagation of lipid peroxidation and generating TP radicals [8]. However, TPs are gradually consumed and converted into a range of oxidation products during antioxidation, among which tocopherol quinones (TQs) are considered important derivatives [9].

As mentioned above, TQs are formed through the oxidation of the chromanol structure of TPs, in which the hydroxyl group is converted into a quinone moiety while the phytyl side chain retains intact [10]. Corresponding to the four TP homologues, TQs also exist as α-, β-, γ-, and δ-TQ [10]. Due to their structural relationship with TPs, TQs are not only regarded as oxidation products of vitamin E, but are also closely linked to the oxidative status of edible oils. In recent years, there has been growing attention to the biological and chemical relevance of TQs. For instance, α-TQ has been reported to modulate claudins and enhance intestinal epithelial tight junction function via the AhR and Nrf_2_ pathways [11]. Additionally, TQs may participate in antioxidant interactions with synergistic compounds such as phospholipids, ascorbyl derivatives, or Maillard reaction products, contributing to the regeneration of TPs [12,13]. γ-TQ and α-TQ has also been detected during frying in canola oil, indicating that they form under real food processing conditions [14]. Conversely, certain TQ homologues, especially γ- and δ-TQ, have been reported to exhibit cytotoxic effects, likely due to their electrophilic properties [15,16]. Chronic exposure to TQs may also be associated with impaired DNA repair and increased mutagenic risk [17]. These findings indicate that monitoring both TP depletion and TQ formation is essential for a comprehensive evaluation of oxidative changes in oil systems.

Given the close relationship between TPs and TQs, their simultaneous determination is highly significant for assessing oil quality and oxidation status. However, analytical studies on TP and TQ remain limited, and most existing methods determine these compounds separately [5]. One challenge is that authentic TQ standards are not readily available commercially, which hinders method development and validation for simultaneous determination of TP and TQ homologues. This often requires different chromatographic conditions, detectors, or sample preparation procedures, increasing analytical complexity and reducing efficiency. Normal-phase high-performance liquid chromatography (NP-HPLC) has been widely applied for TP analysis due to its strong capability to separate structurally similar tocopherol homologues. Nevertheless, most reported methods focus on TP homologues and often fail to achieve satisfactory simultaneous discrimination of individual TQ homologues [18]. Reverse-phase HPLC (RP-HPLC) has also been used for TP and TQ analysis. However, it frequently requires pretreatment steps such as saponification, which are labor-intensive and may cause analyte loss [19]. Additionally, while fluorescence detection is highly sensitive for TP homologues, it is generally less suitable for TQ determination, making integrated analysis of both compound classes challenging [20,21]. Previous studies have reviewed the identification and analysis of TQ, including γ-TQ and related oxidation products [5]. Liquid chromatography-mass spectrometry (LC-MS) offers high structural selectivity but involves expensive instrumentation and complex operation. To date, simple HPLC methods capable of simultaneously separating and quantifying four TPs and their corresponding four TQ homologues under a single analytical condition remain limited.

Thus, these analytical challenges highlight the need for a reliable and simplified method for simultaneous determination of TP and TQ homologues. In this work, a normal-phase HPLC method equipped with a UV spectrophotometric detector (NP-HPLC-SPD) was developed for the simultaneous analysis of four TP and TQ homologues under a single chromatographic condition. Compared with previous method that either focused on TP homologues or required separate analytical procedures for TP and TQ, the present method provides an integrated strategy for resolving both TP and TQ homologues. The methodological value of this approach is reflected in its ability to separate eight structurally related TP and TQ homologues within a short analysis time without laborious pretreatment such as saponification, thereby reducing analytical complexity and potential analyte loss. Chromatographic variables were systematically optimized to improve analyte resolution, and the method was validated for linearity, sensitivity, and precision in accordance with ICH Q2(R1). Recovery experiments were also conducted in selected edible oil matrices to further assess method applicability. Finally, the validated method was applied to peanut oil and camellia seed oil samples. This study provides a practical analytical strategy for integrated determination of TP and TQ in edible oils and facilitates evaluation of TP oxidation in vegetable oil matrices.

## 2. Materials and Methods

### 2.1. Chemicals

*n*-Hexane, ethanol, FeCl_3_, and NaSO_4_ of analytical grade were obtained from Sinopharm Co., Ltd. (Shanghai, China). Standards of α-, β-,γ-,δ-tocopherols (α-, β-,γ-,δ-TP, ≥98%), as well as α-tocopherol quinone (α-TQ, ≥97%), were purchased from Aladdin Reagents Co., Ltd. (Shanghai, China). β-tocopherol quinone (β-TQ), γ-tocopherol quinone (γ-TQ), and δ-tocopherol quinone (δ-TQ) were synthesized and purified in the laboratory. *n*-Hexane and isopropanol of pure chromatography grade were purchased from J&K Chemical Technology Co., Ltd. (Shanghai, China). Meanwhile, Tetrahydrofuran of pure chromatography grade was purchased from Fisher Chemical (Waltham, MA, USA). Peanut oil and camellia oil were purchased from the supermarket (Wuhu, China).

### 2.2. Synthesis of TQ Samples

Only α-TQ is commercially available, whereas β-, γ-, and δ-TQ are not readily obtainable. Therefore, these three TQs were synthesized in our laboratory. Briefly, the synthesis was performed according to the method of Kresp with minor modifications [14]. For β-TQ, 40 mL of β-TP in ethanol (0.5 mg/mL) was mixed with 20 mL of ferric chloride in ethanol (3.5 mg/mL) and reacted at 55 °C for 15 min under high-speed stirring. The reaction mixture was then transferred to a separatory funnel, followed by the addition of 100 mL *n*-hexane and 100 mL water. After vigorous shaking, the aqueous phase was discarded, and the remaining *n*-hexane phase was dried over anhydrous sodium sulfate and concentrated by rotary evaporation. γ-TQ and δ-TQ were synthesized using a similar procedure in methanol, yielding the corresponding crude products. All samples were stored at −20 °C prior to purification.

### 2.3. Separation and Purification of TQ

To improve the purity of the synthesized TQs, further purification was conducted following the method by Kresp [14] with minor modifications. Briefly, α-, β-, γ-, and δ-TQ crude products were purified by column chromatography using a glass column (2.0 cm i.d.) packed with a 40 g silica gel (38–75 μm). Elution was conducted with *n*-hexane/diethyl ether using the following stepwise solvent program: 50 mL (95:5, *v*/*v*), 100 mL (90:10, *v*/*v*), 100 mL (80:20, *v*/*v*), 100 mL (60:40, *v*/*v*), and 100 mL (50:50, *v*/*v*). After purification, the purity of α-, β-, γ-, and δ-TQ reached up to 95%, 96%, 85%, and 81%, respectively, as determined by HPLC. Purified samples were transferred to amber vials and stored at −20 °C.

### 2.4. Preparation of TP and TQ Sample Solutions

A certain amount of four TP standards (α-, β-, γ-, and δ-TP) and four TQ purities/standards (α-, β-, γ-, and δ-TQ) were accurately weighed. Each substance was dissolved in *n*-hexane and made up to a final volume of 5 mL volumetric flask to obtain stock solutions. Aliquots of 0.125, 0.25, 0.375, 0.50, and 0.625 mL of each stock solution were then accurately pipetted, combined, and diluted to 5 mL with *n*-hexane to prepare mixed standard solutions at five concentration levels. The mixtures were vortexed for 30 s to ensure homogeneity, transferred to amber vials, sealed, and stored at −20 °C for HPLC analysis. The nominal concentrations of synthesized TQ standards were corrected according to their HPLC-determined purities before preparation of calibration curves.

### 2.5. Optimization of the Mobile Phase Composition

Mobile phase optimization was carried out with reference to the multicomponent separation strategy reported by Kreps [22]. Different combinations of *n*-hexane, tetrahydrofuran, and isopropanol were tested, and chromatographic performance was evaluated in terms of retention behavior and peak resolution. Four elution conditions were evaluated:

(a) Condition A: Isopropanol as phase A and *n*-hexane as phase B (A:B = 98.8:1.2, *v*/*v*), flow rate of 1.0 mL/min.
(b) Condition B: *n*-Hexane: isopropanol (98.8:1.2, *v*/*v*), flow rate 0.8 mL/min.
(c) Condition C: Same composition as Condition A, flow rate 0.8 mL/min.
(d) Condition D: Phase A: isopropanol; Phase B: *n*-hexane/tetrahydrofuran (96:4, *v*/*v*), A:B = 98.8:1.2 (*v*/*v*), flow rate 0.8 mL/min.

The final chromatographic condition was selected based on the effective discrimination of the eight analytes within 15 min.

TP and TQ homologues were determined using an NP-HPLC system coupled to an SPD (Shimadzu Corporation, Kyoto, Japan). Separation was achieved on a normal-phase Sepax HP-Silica column (4.6 mm × 250 mm, 5 μm, 120 Å; Sepax Technologies, Inc., Newark, DE, USA) under the selected elution condition. The injection volume was 20 μL, and UV detection was performed at 268 nm. After membrane filtration, samples were introduced by an autosampler for the simultaneous determination of four TP and four TQ homologues.

### 2.6. Method Validation Procedures

For validation, mixed standard solutions containing TP and TQ were prepared at five concentration levels were analyzed using the external standard approach to generate calibration curves. Analytical performance was assessed in terms of linearity, sensitivity, precision, and accuracy. Linearity was considered acceptable when the correlation coefficient (R^2^) exceeded 0.9800. Detection and quantification capabilities of the method were evaluated according to ICH Q2(R1), with the limit of detection (LOD) and limit of quantification (LOQ) calculated as LOD = 3σ/S and LOQ = 10σ/S, where σ is the standard deviation of the response obtained from repeated low-concentration measurements and S is the slope of the corresponding calibration curve [23].

Repeatability was evaluated at the intra-day and inter-day levels. Intra-day precision was assessed by six replicate measurements on the same day by the same operator using the same instrument, while inter-day precision was determined from three replicate measurements on three different days. Precision was expressed as relative standard deviation (RSD) for each sample. Recovery experiments were performed in peanut oil and camellia oil by spiking known amounts of TP and TQ mixed standards prior to analysis [24].

$$Recovery(\%)=\frac{Measured\;concentration\;after\;spiking-Original\;concentration}{spiked\;concentration}\;\times \;100\%$$

### 2.7. Analysis of TP and TQ in Oil Samples

Samples of peanut oil and camellia oil obtained from different commercial sources were analyzed. Briefly, each oil sample (approximate 200 mg) was weighed into a centrifuge tube and mixed with *n*-hexane (2 mL). The mixture was vortexed for 2 min until fully dissolved. After thorough mixing, 1 mL of the resulting solution was filtered through a 0.22 μm organic phase membrane filter prior to HPLC analysis. The filtrate was then injected into the HPLC system.

### 2.8. Statistical Analysis

Analysis of variance (ANOVA) was used to compare variables, and Duncan’s multiple range tests were performed to identify significant differences among means (*p* < 0.05), using SPSS version 19.0 for Windows (SPSS Inc., Chicago, IL, USA). Data were analyzed by using LabSolutions 2023 (La Jolla, CA, USA), Excel 2025 (Microsoft Corporation, Redmond, WA, USA), and Origin 2025 (OriginLab Corporation, Northampton, MA, USA).

## 3. Results and Discussion

### 3.1. Synthesis and Determination of the Purity of TQ

According to Section 2.2, ethanol or methanol is preferred as the solvent system for TQ synthesis from TP. These solvents possess moderate polarity, which allows effective dissolution of TP (approximately 50–80 g/L at 25 °C) and partial dissolution of ferric chloride (about 70 g/L in methanol and 30 g/L in ethanol), thereby enabling the reaction to proceed in a homogeneous system. Such homogeneity is essential for efficient and uniform oxidation.

Based on preliminary optimization, a reaction temperature of 50–60 °C and a ferric chloride dosage of approximately 3–3.5 times the mass of TP was considered suitable for TQ synthesis. Excessively high temperatures or excessively high concentrations of FeCl_3_ will lead to the formation of excessive oxidation by-products. Conversely, low temperatures and insufficient FeCl_3_ dosage are unfavorable for the complete oxidation of TP. Consequently, a 55 °C water bath for 15 min was selected as the optimal heating condition. After the oxidation reaction, TLC plates were employed to identify the four types of TQs. However, the purity obtained through this preliminary step remained insufficient, necessitating further purification.

**Figure 1:**
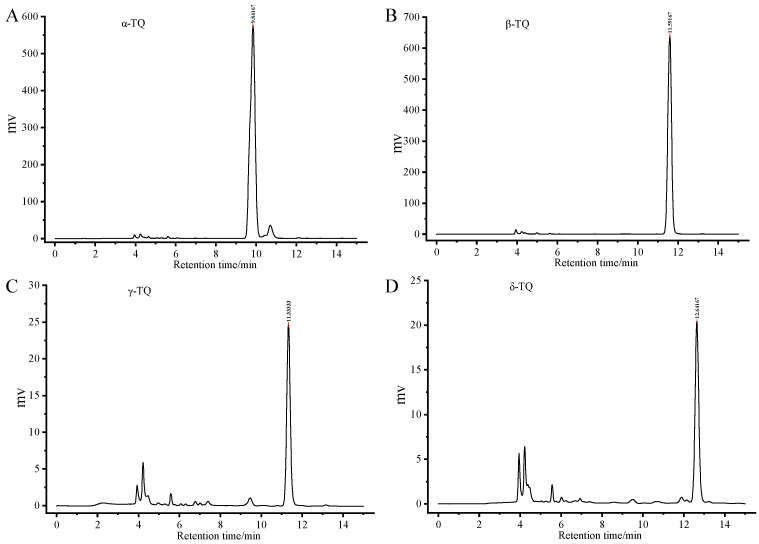
Chromatogram of α-TQ (**A**), β-TQ (**B**), γ-TQ (**C**), and δ-TQ (**D**) standard with HPLC-SPD.

The crude TQ products were initially assessed by HPLC analysis, revealing an approximate purity of 85% for α-TQ and 60–70% for β-, γ-, and δ-TQs. Column chromatography was subsequently applied for further separation and purification [25], which markedly improved the purity of the TQ fractions. The chromatographic profiles of the purified TQ fractions are presented in Figure 1. Based on HPLC peak area normalization excluding the early solvent and interference region, the purities of α-TQ and β-TQ exceeded 95%, while those of γ-TQ and δ-TQ were above 85% and 80%, respectively. The identities of the synthesized TQ compounds had been previously confirmed by LC–MS in our laboratory [5]. Therefore, structural characterization was not repeated in this present study.

### 3.2. Development of Simultaneous Determination of TP and TQ Homologues

**Figure 2:**
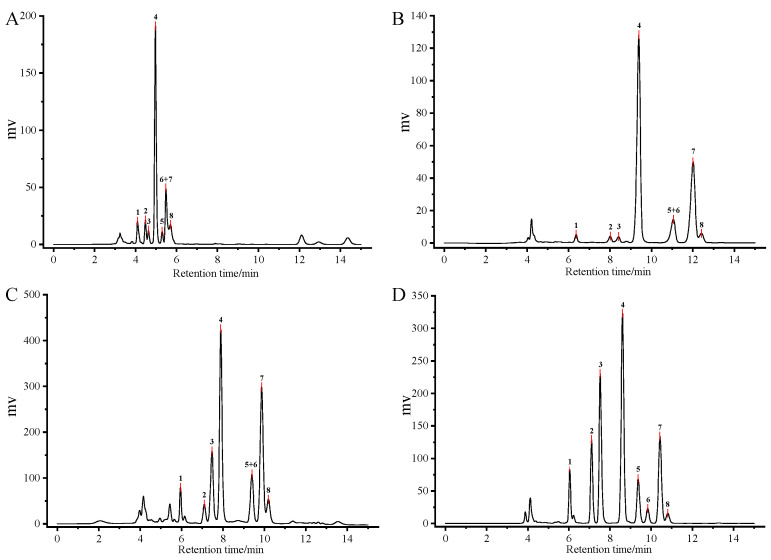
Chromatogram of the mixed solution under different conditions (**A**–**D**). (For Condition (**D**), peak 1: α-TP, peak 2: β-TP, peak 3: γ-TP, peak 4: α-TQ, peak 5: δ-TP, peak 6: γ-TQ, peak 7: β-TQ, peak 8: δ-TQ).

In this study, NP-HPLC was employed for the simultaneous analysis of TP and TQ isomers on a conventional silica column (4.6 mm × 250 mm, 5 μm). Different mobile-phase systems consisting of *n*-hexane, tetrahydrofuran, and isopropanol were evaluated at flow rates of 0.8–1.0 mL/min. Four representative conditions demonstrating effective separation were selected for further comparison. The corresponding chromatograms obtained under these conditions are shown in Figure 2.

In terms of separation performance, Condition A allowed the detection of multiple components but failed to achieve baseline separation of the four TP and four TQ homologues. For example, β-TQ and γ-TQ eluted at approximately 5.486 min, and the resolution (Rs) between adjacent peaks was generally insufficient. Under Condition B, reducing the flow rate altered the retention behavior and improved resolution to some extent. However, overlap between δ-TP and γ-TQ was still observed. Condition C further reduced the retention times (RT) for TP and TQ homologues, and improved peak resolution. In Condition D, increasing the proportion of tetrahydrofuran did not markedly affect the retention of TP homologues but shifted the elution of γ-, β-, and δ-TQ. This adjustment prevented co-elution of δ-TP with γ-TQ and improved the separation between β-TQ and γ-TQ. Therefore, Condition D was selected as the better separation condition compared to other three conditions for the simultaneous analysis of TP and TQ homologues in vegetable oils.

Quantitative chromatographic parameters further confirmed the superiority of Condition D (Table 1). Under Conditions A–C, several representative target peaks exhibited Rs values below 1.5, indicating incomplete separation or close elution of adjacent peaks. While Conditions B and C showed some improvement over Condition A, certain critical peaks still displayed insufficient Rs. In contrast, Condition D improved chromatographic separation of the eight target peaks within 15 min, with most Rs values exceeding 1.5 and tailing factors (TF) within acceptable limits except for chromatographic peak 8. If possible, further optimization could be carried out in future work to achieve complete separation of all eight peaks with satisfactory resolution.

Under the optimized Condition D, the elution order was α-TP (6.042 min), β-TP (7.100 min), γ-TP (7.517 min), α-TQ (8.600 min), δ-TP (9.367 min), γ-TQ (9.833 min), β-TQ (10.425 min), and δ-TQ (10.808 min) (Table 1). These results demonstrate that Condition D provided the most suitable balance among chromatographic resolution, peak symmetry, and analysis time, justifying its selection as the optimized condition. The developed method was then applied to the simultaneous determination and quantification of four TPs and four TQ homologues in oil samples.

### 3.3. Method Validation Results

Table 2 and Table 3 summarize the calibration equations and the corresponding validation results, including linearity, intra-day and inter-day precision, LOD, and LOQ.

#### 3.3.1. Linearity, LOD, and LOQ

Previous studies have reported chromatographic methods for the determination of TPs and TQs in biological samples. For instance, Kiyose developed a chiral HPLC-UV method for the simultaneous separation and determination of RRR- and SRR-α-TP and their quinones in rat plasma and tissues [26]. Subsequently, RP-HPLC-UV methods were established for the simultaneous determination of α-TP and γ-TP and their quinones [14,25]. Although these methods demonstrated satisfactory separation and linearity, neither study reported the limits of detection or quantification, which may limit their applicability for trace-level analysis of TQ homologues. Moreover, these studies mainly focused on selected TP or TQ species rather than the simultaneous determination of four TP and four TQ homologues. Therefore, the analytical coverage and validation completeness of previous methods remain limited for comprehensive TP and TQ profiling.

Under Condition D, the calibration curves of all TP homologues showed acceptable linearity over the studied concentration ranges, with R^2^ values ranging from 0.9850 to 0.9996. The method provided LODs of 0.140–0.371 μg/mL for TP homologues and 0.0564–0.0856 μg/mL for TQ homologues, with corresponding LOQs of 0.467–1.235 μg/mL and 0.1708–0.2595 μg/mL, respectively (Table 2). Here, we would like to point out that the γ-TQ and δ-TQ currently prepared displayed relatively lower purities compared with commercially available reference standards. In future work, we will further optimize the purification procedures and parameters to improve the purity of these TQs used for standard curve establishment, aiming to meet the standard requirement for reference materials with purities above 95%.

The sensitivity of the proposed method for the TP isoforms was further evaluated by comparison with previously reported HPLC methods for TP determination. The results indicated that the sensitivity obtained in this study was generally consistent with literature values for HPLC–UV methods. For example, NP- or RP-HPLC methods coupled with UV or DAD detection typically report LODs of 0.2–0.6 mg/kg for TP homologues, such as 0.38 mg/kg reported by Arisa [27] and 0.20–0.35 mg/kg reported by Yuan [28]. In contrast, methods employing fluorescence detection achieve substantially lower LOD (< 0.05 μg/mL) [29]. However, fluorescence-based methods are less suitable for TQ analysis due to the inherently low fluorescence response of quinone structures. Therefore, although UV-based methods exhibit slightly higher detection limits than fluorescence detector (FLD)-based approaches, they provide a more balanced and practical solution for the simultaneous analysis of TP and TQ homologues.

For TQ homologues, although several studies have reported the simultaneous analysis of selected TQ species, such as α-, β-, and γ-TQs [19], few existing HPLC methods have reported the simultaneous determination of all four TQ homologues. Furthermore, the limits of detection for individual TQ homologues have rarely been reported in the literature. To the best of our knowledge, few studies have reported a systematic validation of a NP-HPLC method covering four TQ homologues together with their corresponding TP homologues, including calibration equations, LODs, LOQs, and precision parameters for each analyte.

#### 3.3.2. Precision of TP and TQ

Method precision and accuracy were evaluated at three concentration levels based on the relative standard deviations (RSD) of intra-day and inter-day measurements (Table 3). The developed NP-HPLC method showed good repeatability and intermediate precision for four TP and TQ homologues at 10, 15, and 25 μg/mL. Overall, the intra-day and inter-day RSDs ranged from 0.49% to 2.69% and from 0.44% to 3.78%, respectively, indicating stable instrument response and reliable peak integration.

Notably, a slightly higher inter-day RSD was observed for γ-TQ at the lowest concentration level, 3.78% at 10 μg/mL, which may be related to the lower signal-to-noise ratio and increased sensitivity to baseline fluctuation. Nevertheless, all RSD values remained within an acceptable range for quantitative analysis. Collectively, these results demonstrate that the proposed NP-HPLC method provides reliable precision for the simultaneous determination of TP and TQ homologues in neat solutions.

In comparison, broader precision ranges are often observed in complex food matrices. For example, an NP-HPLC-UV/Vis method for TP isoforms in various food matrices reported precision values below 15%, highlighting the pronounced impact of matrix complexity on analytical variability [27]. However, this method mainly focused on TP isoforms and did not include the simultaneous determination of TQ. While LC-MS methods offer higher structural selectivity and sensitivity for TP and TQ homologues, they require more expensive instrumentation and complex operation. Therefore, the present NP-HPLC-SPD method is not intended to replace LC-MS approaches but to provide a simpler and more accessible strategy for routine simultaneous analysis of TP and TQ homologues in edible oil matrices [30,31].

Comparable precision has also been reported for methods targeting α-TP together with α-TQ. Tang [30] reported that simultaneous analysis of α-TP and α-TQ by UPLC-Q-TOF-MS achieved repeatability and intermediate precision values of less than 1.82% RSD (*n* = 6) and 3.55% RSD (*n* = 3), respectively. Although this MS-based method showed good precision, showed excellent precision, it mainly focused on selected α-TP rather than multiple TP and TQ homologues. Broader precision ranges have also been reported for multi-analyte vitamin E workflows incorporating α-TQ in biological matrices. For instance, an NP-LC-APCI-MS method reported intra-day precision of 2–17% and inter-day precision of 5–18% across analytes and levels, suggesting that increased method complexity and matrix effects can substantially elevate variability [31]. Earlier HPLC- electrochemical detection (ECD) work also demonstrated the feasibility of simultaneous α-TP and α-TQ detection in biological samples [32], whereas studies in erythrocytes reported inter-day RSDs of approximately 6%, further illustrating the influence of complex matrices and sample handling on precision [33]. These comparisons indicate that previously reported methods may exhibit broader RSD ranges when applied to complex matrices or multi-analyte workflows, and most were limited to TP homologues, α-TP and α-TQ, or selected oxidation products rather than the simultaneous evaluation of four TP and TQ homologues. These results suggest that the proposed NP-HPLC method offers reliable precision for simultaneous TP and TQ isomer determination in a neat solvent system. Compared with the reported methods discussed above, the present method provides a relatively simple chromatographic approach for evaluating multiple structurally related TP and TQ homologues under the same analytical conditions.

However, it should be emphasized that these validation data were obtained using mixed standards prepared in *n*-hexane, where the sample composition is simple and largely free of matrix-related effects. To further assess the method in real oil matrices, recovery experiments were subsequently performed in peanut oil and camellia oil.

#### 3.3.3. Assessment of Recovery of TP and TQ Homologues in Oil Matrices

To further evaluate the analytical performance of the proposed method in real oil matrices, the recoveries of TP and TQ homologues in camellia oil and peanut oil were determined, as shown in Table 4.

The recoveries of TP and TQ homologues in peanut oil and camellia oil confirmed the acceptable analytical accuracy of the proposed NP-HPLC method in real oil matrices. For TP homologues, the recoveries ranged from 91.7 ± 1.7% to 105.8 ± 1.2% in peanut oil and from 90.9 ± 1.6% to 97.7 ± 1.0% in camellia oil. For TQ homologues, the corresponding ranges were 82.8 ± 2.4% to 98.7 ± 0.9% and 79.5 ± 2.0% to 101.8 ± 1.1%, respectively. In general, TP homologues exhibited relatively high recoveries in both oil matrices, whereas TQ homologues showed slightly greater variation, particularly for β-TQ and δ-TQ. Nevertheless, all analytes showed recoveries within an acceptable range, indicating that the proposed NP-HPLC method could provide satisfactory accuracy and repeatability for the simultaneous analysis of TP and TQ homologues in edible oils. Although these recovery results supported the applicability of the proposed method in selected edible oil matrices, broader matrix validation would still be valuable to further evaluate matrix effects and quantitative robustness across more diverse oil systems. The recovery experiments were conducted using only a single spiking level and two oil matrices. Therefore, these results should be regarded as an initial assessment of matrix applicability rather than a comprehensive matrix validation. Broader matrix validation using multiple spiking levels and more diverse oil systems would still be valuable to further evaluate matrix effects and quantitative robustness.

### 3.4. TP and TQ in Oil Samples

To determine the detectable TP and TQ homologues in peanut oil and camellia oil, a direct dissolution normal-phase chromatographic method was employed to minimize potential losses of target compounds during complex pretreatment procedures. It should be noted that all oils analyzed in this study were commercially obtained directly from different batches. The contents of TP and TQ homologues in each oil sample are summarized in Table 5. However, the contents of β-TP and δ-TP in the samples were extremely low or below the method detection limits, and their corresponding oxidation products were also not detected. Therefore, they are not included in Table 5 [24].

Across different batches of vegetable oils, TP and TQ levels showed some variability, and the compositional profiles differed markedly between peanut oil and camellia oil. In peanut oil, α-TP and γ-TP were detected, with γ-TP present at higher levels than α-TP, indicating that α-TP and γ-TP were the predominant detectable TP components in these samples. This distribution aligns with the typical TP profile reported for peanut oil [34]. Among the three batches, TP levels showed relatively small variations, whereas TQ, particularly γ-TQ, displayed more pronounced differences. Notably, peanut oil 2 exhibited a markedly higher γ-TQ level than the other two batches, which may be attributed to variations in storage conditions, light exposure, thermal history, or overall oxidation status, as these factors can accelerate TP oxidation and subsequent TQ formation [18,19,20]. In camellia oil, α-TP was the only detectable TP, and its level was markedly higher than that in peanut oil, indicating that α-TP is the major TP component in camellia oil, consistent with previous studies [35,36]. The α-TP content varied among batches, with camellia seed oil 3 showing a relatively lower α-TP level. In contrast, camellia oils 1 and 2 exhibited relatively high α-TQ levels, whereas both α-TQ and γ-TQ were low in camellia oil 3, reflecting substantial differences in oxidative status among batches. The elevated α-TQ levels in oils 1 and 2 may be associated with prior storage or processing conditions; however, the specific causes could not be determined due to the commercial nature of the samples.

## 4. Conclusions

In summary, a reliable NP-HPLC method coupled with UV detection (SPD) was developed for the simultaneous determination of TP and TQ homologues. Under optimized chromatographic conditions, eight target compounds were effectively separated within 15 min, exhibiting good linearity, satisfactory resolution, and acceptable limits of detection and quantification. Validation in standard solutions, along with recovery experiments in peanut oil and camellia oil, confirmed the applicability of the method in selected edible oil matrices. Application of the method to commercial oil samples revealed clear differences in TP composition between peanut and camellia oils, while TQ homologues were detected in some batches, reflecting variations in oxidation status. Overall, the proposed method provides a useful analytical basis for the simultaneous separation and determination of TP and TQ homologues inedible oil.

Nevertheless, broader validation across diverse oil matrices, including matrix-matched calibration and robustness testing, would strengthen the method’s applicability for routine analysis. Future studies under controlled storage, light exposure, and thermal processing conditions could further elucidate the formation of TQ homologues and the oxidative conversion of TP in vegetable oils. In addition, signal enhancement strategies, such as fluorescence derivatization, may be explored to improve TQ detection sensitivity, enabling trace-level analysis in more complex oil matrices.

## Figures and Tables

**Table 1:** Quantitative chromatographic parameters of target peaks under Conditions A–D.

Condition	Peak	RT/min	Rs	TF
A	1	4.100	1.192	1.580
	2	4.483	1.934	—
	3	4.633	0.871	—
	4	4.975	2.023	1.122
	5	5.300	1.853	—
	6	5.483	1.005	—
	7	5.708	0.727	—
B	1	6.367	2.600	1.113
	2	8.017	1.581	—
	3	8.417	1.342	—
	4	9.367	1.680	0.879
	5	11.058	4.646	0.812
	6	12.008	2.338	0.893
	7	12.417	0.923	—
C	1	5.950	1.294	—
	2	7.100	1.119	—
	3	7.467	1.418	0.985
	4	7.892	1.684	1.062
	5	9.400	1.709	0.932
	6	9.867	1.543	1.086
	7	10.192	1.034	—
D	1	6.042	2.205	1.107
	2	7.100	3.946	1.100
	3	7.517	1.935	1.204
	4	8.600	2.148	1.045
	5	9.367	2.957	1.120
	6	9.833	1.745	0.982
	7	10.425	2.158	1.072
	8	10.808	1.245	—

Note: RT: retention time; Rs: resolution; TF: tailor factor, and peaks (peak 1: α-TP, peak 2: β-TP, peak 3: γ-TP, peak 4: α-TQ, peak 5: δ-TP, peak 6: γ-TQ, peak 7: β-TQ, peak 8: δ-TQ). “—” indicates that the parameter could not be reliably calculated due to incomplete separation or peak overlap.

**Table 2:** Method validation results for the determination of TP and TQ homologues.

Samples	Regression Equation	R^2^	LOD (μg/mL)	LOQ (μg/mL)
α-TQ	y = 57223x − 7201.1	R^2^ = 0.9941	0.0656	0.1987
β-TQ	y = 20731x − 24921	R^2^ = 0.9970	0.0856	0.2595
γ-TQ	y = 8901.6x − 10640	R^2^ = 0.9911	0.0564	0.1708
δ-TQ	y = 6921.8x − 2659.4	R^2^ = 0.9996	0.0760	0.2549
α-TP	y = 1713.4x + 8962.3	R^2^ = 0.9996	0.2710	0.9030
β-TP	y = 1368.9x + 9519.3	R^2^ = 0.9946	0.3710	1.2350
γ-TP	y = 1418.8x + 8595.2	R^2^ = 0.9850	0.1400	0.4670
δ-TP	y = 2392.5x − 30862	R^2^ = 0.9899	0.2540	0.8540

**Table 3:** Precision and Accuracy of TP and TQ.

Samples	Concentration (μg/mL)	Intra-Day RSD	Inter-Day RSD
α-TP	10	0.59%	3.00%
	15	1.50%	2.11%
	25	0.58%	2.18%
β-TP	10	0.50%	3.46%
	15	2.01%	2.89%
	25	1.10%	2.29%
γ-TP	10	2.69%	2.33%
	15	0.62%	3.54%
	25	1.76%	2.75%
δ-TP	10	0.95%	0.94%
	15	1.03%	1.39%
	25	0.96%	1.93%
α-TQ	10	0.50%	1.53%
	15	1.32%	1.48%
	25	0.91%	2.01%
β-TQ	10	0.50%	1.42%
	15	1.17%	1.87%
	25	0.93%	2.22%
γ-TQ	10	0.83%	3.78%
	15	1.47%	3.52%
	25	0.92%	2.28%
δ-TQ	10	0.49%	0.44%
	15	1.37%	2.03%
	25	0.98%	1.77%

**Table 4:** Recoveries of TP and TQ homologues in peanut oil and camellia oil matrices.

Samples	Spiked Level (mg/kg)	Peanut Oil Recovery (%)	Camellia Oil Recovery (%)
α-TP	50	105.8 ± 1.2	97.7 ± 1.0
β-TP	50	94.9 ± 1.5	92.4 ± 1.8
γ-TP	50	96.6 ± 1.1	94.1 ± 1.4
δ-TP	50	91.7 ± 1.7	90.9 ± 1.6
α-TQ	10	98.7 ± 0.9	101.8 ± 1.1
β-TQ	10	87.3 ± 2.1	85.7 ± 2.3
γ-TQ	10	96.4 ± 1.3	92.1 ± 1.7
δ-TQ	10	82.8 ± 2.4	79.5 ± 2.0

Values are expressed as mean ± SD (*n* = 3).

**Table 5:** Contents of detectable TP and TQ homologues in peanut oil and camellia oil samples.

Samples	α-TP (mg/kg)	γ-TP (mg/kg)	α-TQ (mg/kg)	γ-TQ (mg/kg)
Peanut oil 1	143.8 ± 5.2 ^b^	164.2 ± 9.3 ^a^	1.34 ± 0.57 ^b^	0.36 ± 0.18 ^a^
Peanut oil 2	123.0 ± 2.9 ^b^	163.8 ± 4.7 ^a^	2.44 ± 0.10 ^b^	8.44 ± 0.38 ^a^
Peanut oil 3	145.2 ± 15.1 ^b^	161.6 ± 4.4 ^a^	1.35 ± 0.40 ^b^	1.87 ± 0.05 ^a^
Camellia oil 1	358.5 ± 33.2 ^a^	ND	9.27 ± 0.25 ^a^	1.16 ± 0.23 ^a^
Camellia oil 2	345.5 ± 24.7 ^a^	ND	7.86 ± 0.24 ^a^	2.88 ± 0.49 ^a^
Camellia oil 3	300.0 ± 14.1 ^a^	ND	1.18 ± 0.16 ^b^	ND

Note: “ND” means not detected. Different lowercase letters within the same column indicate significant differences among samples (*p* < 0.05).

## Data Availability

The original contributions presented in the study are included in the article; further inquiries can be directed to the corresponding authors.
